# Left Supraclavicular Lymph Node Metastasis from Ovarian Cancer Associated with Papillary Thyroid Microcarcinoma, a Confusing Pathology-Essential Role of Functional Imaging

**DOI:** 10.3390/diagnostics10050270

**Published:** 2020-04-30

**Authors:** Doina Piciu, Alexandru Meșter, Calin Căinap, Elena Bărbuș, Dragos-Stefan Morariu, Andra Piciu

**Affiliations:** 1 Iuliu Hatieganu University of Medicine and Pharmacy, 400012 Cluj-Napoca, Romania; doina.piciu@gmail.com; 2 Department of Endocrine Tumors and Nuclear Medicine, “Prof. Dr. I. Chiricuță” Institute of Oncology, 400015 Cluj-Napoca, Romania; elena.barbus@gmail.com; 3 Department of Oral Health, Iuliu Hatieganu University of Medicine and Pharmacy, 400012 Cluj-Napoca, Romania; 4 Department of Medical Oncology, “Iuliu Hațieganu” University of Medicine and Pharmacy, 400012 Cluj-Napoca, Romania; calincainap2015@gmail.com (C.C.); piciuandra@gmail.com (A.P.); 5 Department of Surgical Oncology, “Iuliu Hațieganu” University of Medicine and Pharmacy, 400012 Cluj-Napoca, Romania; dragosstefanmorariu@gmail.com

**Keywords:** ovarian carcinoma, supraclavicular metastasis, 18F-fludeoxyglucose positron emission tomography-computed tomography, papillary thyroid microcarcinoma

## Abstract

The revolution of imaging in medicine leads to new standards of care, mostly in specialties like oncology, neurology, or endocrinology. We present a review of the literature and a case report of a 62-year-old patient initially treated for a benign gynecologic pathology and followed-up for 7 years clinically, with serologic and with multiple imaging techniques. There is an actual growing use of highly sensitive functional imaging methods, like fluoro-deoxy-glucose (F18-FDG) positron emission tomography/computed tomography (PET/CT) in the evaluation of oncologic pathologies, staging, follow-up, and therapy response monitoring. This is the first case report described in the literature presenting the association of thyroid papillary microcarcinoma (MPTC) and supraclavicular metastasis of ovarian cancer. The study aims to underline the necessity of a complex and careful evaluation of each oncologic patient, due to the unexpected clinical presentation and rare association of diseases, sometimes leading to confusing management.

## 1. Introduction

Ovarian cancer (OC) spreads most frequently through the intra-peritoneal channels in such way that, in the majority of cases, the disease remains at the pelvic and abdominal levels [1,2,3,4,5,6]. Ovarian cancer may also metastasize through lymphatic channels, extremely rarely, in the supraclavicular lymph node (in the so-called Virchow node) [7,8].

Papillary thyroid cancer (PTC) is the most common thyroid malignancy [9,10,11] and is defined as a malignant epithelial tumor. Papillary thyroid microcarcinoma (MPTC) is a PTC with a maximum diameter of < 1 cm. The incidence of MPTC is increasing due to a real increase of the malignant thyroid pathology and also to the improved, more sensitive diagnostic methods [12,13]. Despite its minimal size, MPTC is capable to metastasize frequently in the cervical lymph nodes, less commonly in other sites, and has an excellent prognosis.

The presence of a supraclavicular lymph node in a case of known thyroid carcinoma is highly suggestive of metastasis from the thyroid; it is less probable to consider another cancer to be the source for this metastasis and improbable for it to be from ovarian cancer. In this situation, the most likely clinical decision would be the diagnosis of thyroid cancer with left supraclavicular metastasis and the first therapeutic decision—total thyroidectomy with selective lymphadenectomy. Actually, the left supraclavicular lymph node might be a site of metastatic spread both for MPTC and OC; thus, a clear evaluation of the clinical history of the patient and an extensive imaging protocol need to be applied to avoid a confusing pathology.

## 2. Case Report

We present the case of a 62-year-old Caucasian woman, who underwent a total hysterectomy in 2012, for benign uterine fibroids, which produced heavy periods and pelvic pain. No malignant issues were confirmed at that moment. The patient has signed an informed consent, according to the institutional protocols of the “Prof. Dr. I. Chiricuță” Institute of Oncology Cluj-Napoca, both for medical procedures and the use of medical records in scientific purposes, respecting the confidentiality.

**Figure 1:**
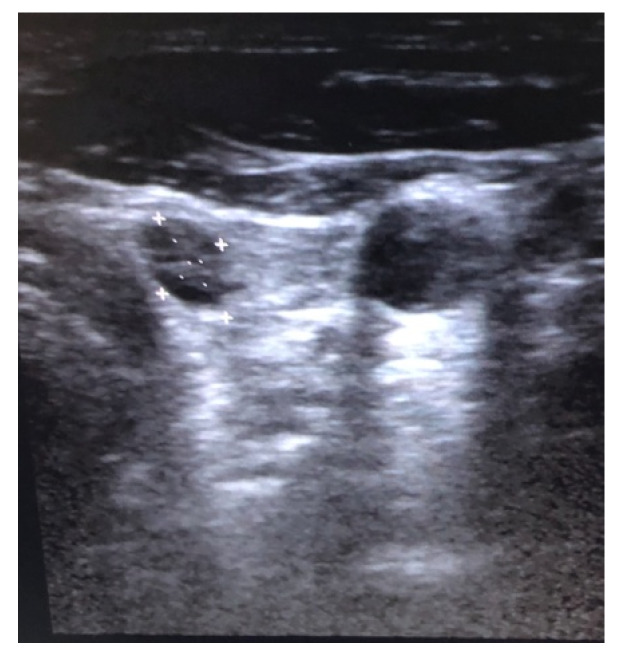
Thyroid ultrasound revealing a left lobe hypoechoic thyroid nodule.

Two years later, the patient was sent to the endocrinologist, because of a lump in the left supraclavicular fossa. The thyroid ultrasound (US) showed a hypoechoic nodule in the left thyroid lobe, measuring 6.9/4.7/3 mm, without microcalcification, but “taller than wider” and with peripheral vascularization (Figure 1). The left supraclavicular lump was consistent for a lymphadenopathy of 18/10.5/9.5 mm with high vascularization, no microcalcification, predominantly hypoechoic relative to the adjacent musculature, round-shaped, and highly suggestive for malignant lymph node. The shape and the intense vascularization of the lymph node were suggestive for malignancy; however, metastatic nodes from papillary carcinoma of the thyroid are usually hyperechoic, and this is believed to be related to the intranodal deposition of thyroglobulin originating from the primary tumor and this also frequently has microcalcifications.

The tumor markers were performed in the same accredited laboratory and consisted in: thyroid-stimulating hormone (TSH, normal values 0.27–4.2 mIU/L), free thyroxine (FT4, normal values 12–22 pmol/L), thyroglobulin antibodies (TgAb, normal values < 115 IU/mL) and thyroglobulin (Tg, normal values < 79 ng/mL), CA125 tumor antigen (normal values < 35 U/mL). All analyses were performed using the same method of electrochemiluminescence, the ECLIA technique.

The thyroid hormone profile was within the normal range with the following values: TSH level was 2.2 mIU/L, in the normal range, FT4—14.1 pmol/L, TgAb—78 IU/mL, and Tg—34 ng/mL. The thyroid hormone evaluation is mandatory in the algorithm of diagnosis of every thyroid nodule. Tg, according to the American Thyroid Association (ATA) guidelines, is not a first-line tumor marker to be assessed in this evaluation, but in the case of metastatic disease, Tg might play a role in orienting the therapeutic plan. The thyroid ultrasound description of left thyroid nodule leads to a classification corresponding to TIRADS 3 [14]; the greater nodule dimension was 6.9 mm (< 1 cm), without significant suspicious signs of malignancy (ex. microcalcification, internal vascularization), the fine-needle aspiration biopsy (FNAB) was indicated as a diagnostic option, according to the American Thyroid Association (ATA) guidelines and European Thyroid Association (ETA) guidelines [9,10]; the procedure was performed and the result, according to the Bethesda System for Reporting Thyroid Cytopathology [15], was Thy 5—suspicious for malignancy. The FNAB result of Thy 5—suspicious for malignancy—in the thyroid nodule and the ultrasound characteristics of the lymph node (round shape, highly hilar and peripheral vascularity, predominantly hypoechoic, not hyperechoic, and without calcification) made us think of an origin of the lymph node metastasis other than the thyroid gland.

Because the left supraclavicular adenopathy was highly suspected for malignancy, but with an atypical ultrasound presentation, comparing with the thyroid nodule, we asked for a F-18 fluorodeoxyglucose (FDG) positron emission tomography/computed tomography (F18-FDG PET/CT).

The F18-FDG PET/CT was performed on a GE Optima 560 equipment, with an activity of 296 MBq of F18-FDG injected intravenously; the images were acquired at 50 min after tracer administration, with the standardized uptake value (SUV) expressed in lean body mass (SUVlbm).

**Figure 2:**
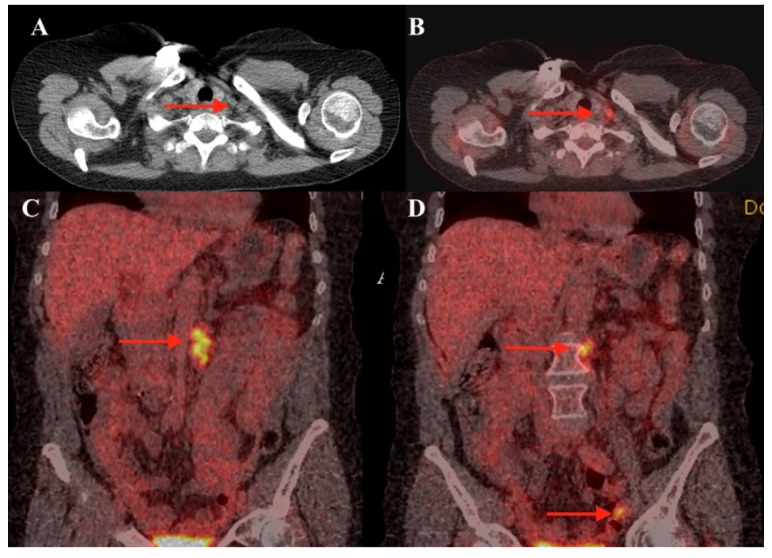
(**A**) F18-fluorodeoxyglucose positron emission tomography/computed tomography (F18-FDG PET/CT) axial CT section cervical level; (**B**) fused PET/CT image axial section at cervical level; (**C**) coronal section PET/CT image at abdominal level; and (**D**) coronal section PET/CT image pelvic level. Red arrows are showing high tracer uptake in the left supraclavicular lymph node, in the left latero-aortic lymph node and the left iliac extern pelvic lymph node.

The baseline F18-FDG PET/CT (Figure 2) was showing a slightly increased radiotracer uptake, suggestive for metastases in the left supraclavicular lymph node (SUVlbm Max—2.64), in the left latero-aortic lymph node (SUVlbm Max—4.72) and the bilateral iliac extern lymph nodes (SUVlbm Max—4.81), and the section in Figure 2 shows only the left one. We found a surprisingly low F18-FDG uptake in the left supraclavicular lymph node, considering the aggressiveness of the tumor and the lymph nodes from the abdomen.

In 2014, a total thyroidectomy with left supraclavicular biopsy was performed, and the pathology report proved a left papillary microcarcinoma classic form, T1aNxMx, and ovarian carcinoma metastasis in the left supraclavicular lymph node. Surprisingly, as the patient underwent a total hysterectomy with both ovaries removed two years before, a second opinion was given on the pathology report of the supraclavicular lymph node specimen, confirming the diagnosis of metastatic ovarian carcinoma. The histopathology specimen from 2012 was revised and the final pathology report was left ovarian carcinoma.

CA 125 is a valuable specific ovarian tumor marker, a fact which imposed its analysis. CA 125 serum level was 932 U/mL, very increased, confirming the status of persistent ovarian carcinoma disease.

The case was considered as multiple malignancies with papillary thyroid microcarcinoma T1aNxMo stage I, low-risk group, and ovarian carcinoma FIGO IVB.

**Figure 3:**
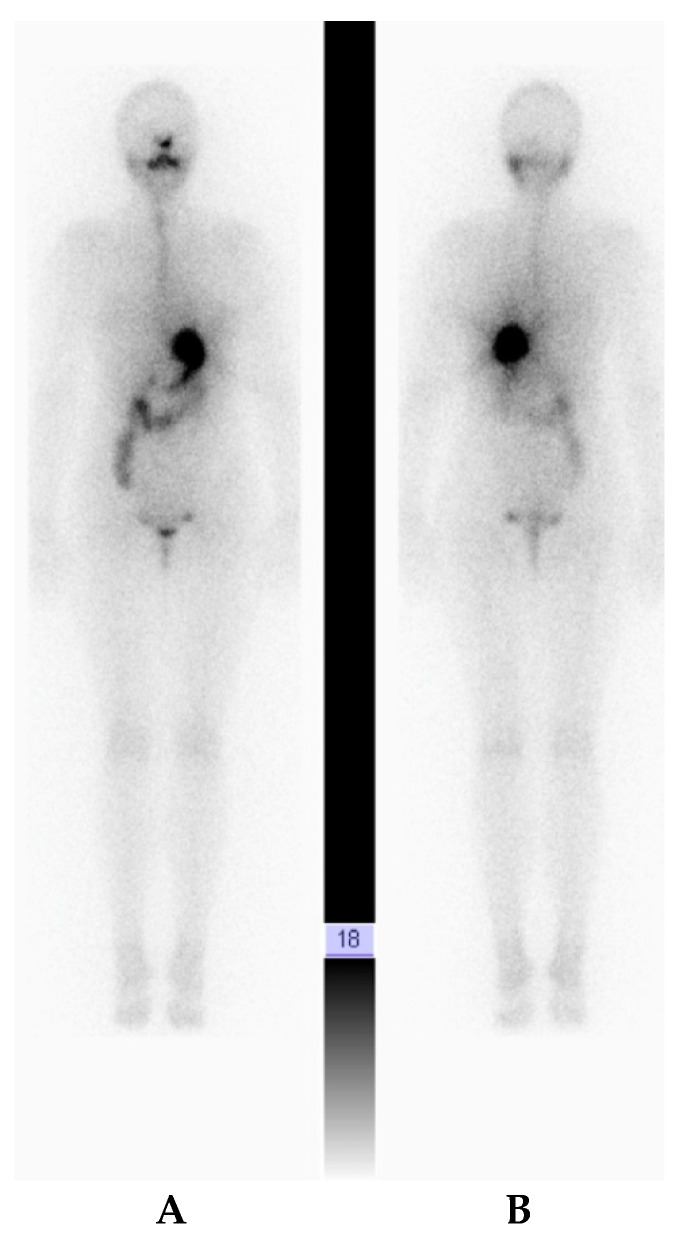
Whole Body Scan with I-131 sodium iodide (I-131 WBS) with 185MBq at 24 h post-administration, showing no thyroid remnant uptake and no abnormal uptake in other sites ((**A**) anterior, (**B**) posterior).

For the thyroid carcinoma, the patient was evaluated after surgery in stimulated conditions, the TSH was 48 IU/mL (increased) and the Tg level was 0.04 ng/mL (undetectable) with TgAb in normal range (TgAb < 10 IU/mL). According to ATA and ETA guidelines [9,10], no radioiodine therapy was necessary; a whole-body scan (WBS) with 185 MBq I-131 was performed to exclude the thyroid origin of the lymph node lesions seen on the PET/CT scan (Figure 3), the WBS being negative. The patient started hormone replacement therapy with 125 micrograms of Levo-thyroxine daily, with TSH and FT4 assessments after 2 months: TSH—0.19 IU/mL and FT4—17.8 pmol/mL; considering that the daily dose was correct and well-tolerated, a mild suppression status of TSH was achieved [16].

The treatment of ovarian cancer consisted of first-line palliative chemotherapy with Paclitaxel 175 m/mp and Carboplatin (AUC5). The tumor marker value decreased at normal values in 04/2016 (CA 125—21 U/mL) and the patient was followed-up by periodic serum CA 125 and chest and abdominal contrast-enhanced CT, showing complete remission.

**Figure 4:**
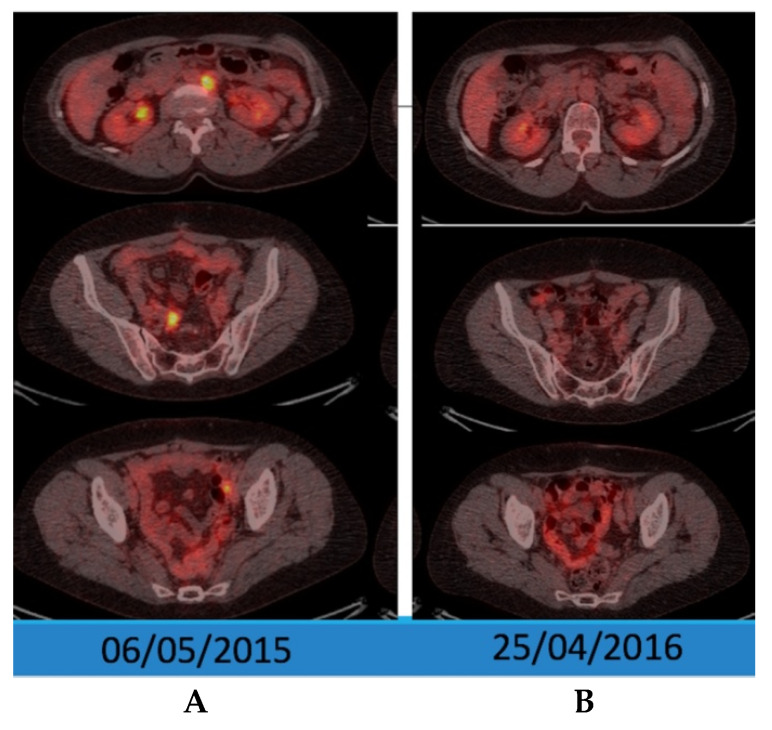
F18-FDG PET/CT scans—2015, before chemotherapy and 2016, after treatment, evaluating the therapy response: axial section abdominal level (upper images), pelvic level with right lymph node (middle image), and pelvic level with left lymph node (lower image), showing tracer high uptake in left abdominal latero-aortic lymph node (SUVlbmMax—5.1), and in bilateral pelvic lymph nodes ((**B**) SUVlbmMax—4.8 and (**A**) SUVlbmMax—3.9), with complete remission in 2016 on all sites.

The therapy response was assessed using CA 125 and F18-FDG PET/CT (Figure 4) showing a complete metabolic remission in 2016 (the third scan) comparative to 2015 (the second scan).

**Figure 5:**
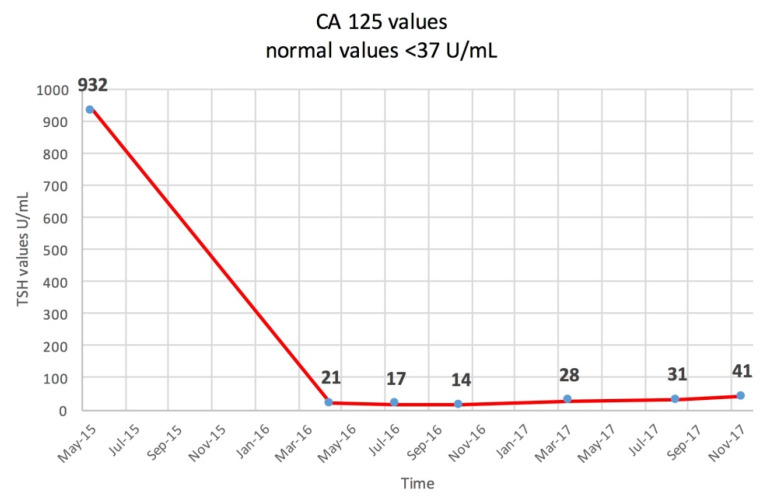
Serum CA 125 levels determined during the follow-up.

**Figure 6:**
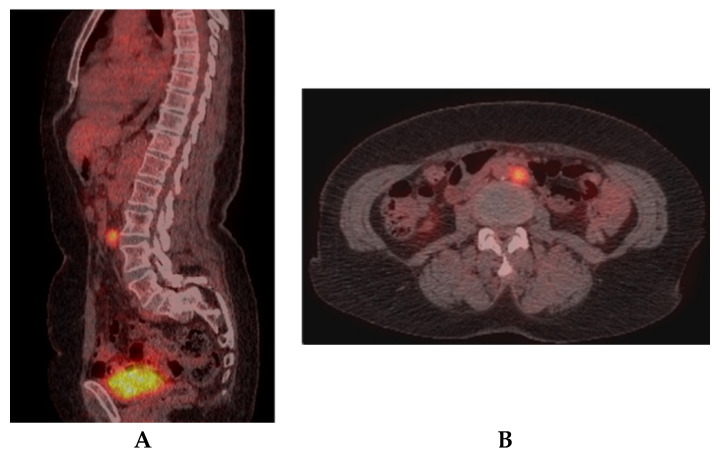
F18-FDG PET/CT scans in 2017 after treatment, evaluating the CA 125 increase during the follow-up: sagittal section abdominal level (**A**), pelvic level (**B**) showing tracer high uptake in left abdominal latero-aortic lymph node (SUVlbm Max—4.43), with recurrence after complete remission in 2016.

The patient continued the follow-up at 3–6 months using clinic, serologic, cervical and abdominal ultrasound, and thorax and abdominal CT, being in complete remission for 1 year. In February 2017, CA 125 rose slightly at 41 U/mL (Figure 5, showing the CA 125 evolution) and another F18-FDG PET/CT, the fourth one during the disease, asked by the oncologist, is shown in Figure 6. Meanwhile, the thyroid carcinoma was declared in complete remission: clinical, Tg undetectable in stimulated conditions (TSH > 35 IU/mL), negative TgAb, and negative I-131 WBS.

Second-line chemotherapy was debuted in 2017, with the same platinum regimen, as the patient presented a disease-free interval of more than a year, considering that it was a platinum-sensitive tumor, followed by maintenance therapy with PARP-inhibitor, as BRCA 1/2 was present. Therapy was administered according to the guidelines and treatment availability at that time [17].

## 3. Discussion

An online search on the PubMed database was performed using the following terms: “ovarian”, “cancer”, “supraclavicular”, “lymph node”, and “metastasis”. In total, 35 articles (case reports and reviews) were obtained. After applying the filters “full text” and “humans”, we had a selection of 17 articles [18,19,20,21,22,23,24,25,26,27,28,29,30,31,32,33,34]. Among these articles, there were only four referring to F18-FDG PET/CT evaluation [18,25,26,29]. According to all the mentioned studies which were analyzed, the extra-abdominal spread of ovarian cancer is rare in the clinical scenario, but very challenging. Data from the literature concerning extra-abdominal lymph node involvement are very poor, thus the interest of studying this was justified. Fanti et al. [29] were among the first who speculated that F18-FDG PET/CT might be a useful tool to detect unusual extra-abdominal nodal involvement in this pathology. In the latest years, the researchers underlined that the method is mandatory to be applied mainly if there is any suspicion of persistent or recurrent disease [18].

According to the recommendation provided by the NCCN Clinical Practice Guidelines in Oncology (NCCN Guidelines) for ovarian cancer Version 1.2020 [17], PET/CT is indicated during the initial evaluation, monitoring/follow-up (chest/abdominal/pelvic CT, MRI, PET/CT, or PET as clinically indicated), and for recurrent disease in any stages after primary treatment in the following situations: rising CA-25 with no previous chemotherapy, clinical relapse with no previous chemotherapy, clinical relapse after previous chemotherapy, and serially rising CA-125 values after previously administered chemotherapy. On the other hand, this imaging method is routinely recommended in the evaluation of malignant thyroid pathology [35,36,37].

In patients with ovarian cancer, the importance of extensively imaging follow-up of the left supraclavicular seems to be beneficial to discover occult disease, and the possible sites of tumor marker CA 125 rises.

In 2010, Bilici et al. [38] published their work referring to the impact of F18-FDG PET/CT on the management of patients with suspected recurrent ovarian carcinoma. The results showed that the sensitivity of FDG PET/CT in detecting the recurrent disease was 95.5% compared with 55.5% for CT scans; the specificity was 93.3% for PET/CT and 66.6% for CT, and the accuracy was 95% for PET/CT and 58.3% for CT scans. Similarly, Gu et al. [39] published a meta-analysis comparing the value of CA 125, PET alone, PET/CT, CT, and MRI in diagnosing of recurrent ovarian carcinoma. The authors reviewed 34 studies and concluded that CA 125 had the highest pooled specificity of 0.93 (95% CI) and PET/CT had the highest pooled sensitivity of 0.91 (95% CI: 0.88–0.94). They concluded that PET/CT might be a useful supplement to the current surveillance techniques, particularly for those patients with an increasing CA 125 level and negative CT or MRI.

Caobelli et al. published [40], in 2016, that in the same FIGO groups the progression-free survival and the overall survival were significantly lower at 2 years and at 4 years, respectively, for FIGO I–II with PET/CT positive, compared with the groups with PET/CT negative, similarly as for FIGO groups III–IV.

F18-FDG PET/CT is more accurate than CT and MRI identification of extra-abdominal metastatic lymphadenopathy. Mediastinal metastatic lymphadenopathies were associated with higher mortality. A preoperative PET/CT in patients with advanced ovarian cancer may alter therapy, direct surgery, and provide a baseline for subsequent treatment monitoring.

Functional imaging using F18-FDG PET/CT succeeded in this case to be an essential tool in the differential diagnosis of the thyroid pathology, in the correct assessment of primary cancer, to evaluate the treatment response, and to change to another strategy, when the relapse was observed.

## 4. Conclusions

According to the literature search, this is the first case report of an association of MPTC and supraclavicular metastasis of ovarian cancer. The study has the aim to underline the necessity of complex and careful evaluation of each oncologic patient, due to the unexpected clinical presentation and rare association of diseases, sometimes leading to a confusing management. The growing evidence available for functional imaging, as F18-FDG PET/CT, in onco-gynaecology will impose new standards both in the initial diagnostic, staging, and in the imaging-based criteria for noninvasive response evaluation.
